# Anti-Inflammatory Activity and Chemical Analysis of Different Fractions from *Solidago chilensis* Inflorescence

**DOI:** 10.1155/2021/7612380

**Published:** 2021-10-29

**Authors:** Thais Morais de Brito, Fabio Coelho Amendoeira, Temistocles Barroso de Oliveira, Laís Higino Doro, Esdras Barbosa Garcia, Naína Monsores Felix da Silva, Amanda da Silva Chaves, Flavia Fontenelle Muylaert, Tatiana Almeida Pádua, Elaine Cruz Rosas, Maria das Graças M. O. Henriques, Valber da Silva Frutuoso, Simone Sacramento Valverde, Fausto Klabund Ferraris

**Affiliations:** ^1^Laboratório de Farmacologia, Instituto Nacional de Controle de Qualidade em Saúde–FIOCRUZ, Rio de Janeiro, Brazil; ^2^Laboratório de Química Medicinal de Produtos Bioativos, Instituto de Tecnologia em Fármacos–FIOCRUZ, Rio de Janeiro, Brazil; ^3^Laboratório de Farmacologia Aplicada, Instituto de Tecnologia em Fármacos–FIOCRUZ, Rio de Janeiro, Brazil; ^4^Laboratório de Imunofarmacologia, Instituto Oswaldo Cruz–FIOCRUZ, Rio de Janeiro, Brazil

## Abstract

*Solidago chilensis* Meyen (Compositae) is a species native to South America (Brazil) popularly known as arnica. In Brazilian popular medicine, inflorescences and rhizomes of this plant have been used since the end of the 19th century to replace the exogenous and hepatotoxic *Arnica montana* L. in the treatment of edema and inflammatory pathologies. Although the anti-inflammatory activity of *S. chilensis* is evidenced in the literature, there is a lack of studies with enriched fractions or compounds isolated from it. The objective of the current study was to characterize phytochemically and to evaluate the pharmacological action *in vivo* and *in vitro* of the crude extract and the different fractions (hexane, dichloromethane, acetal, butanolic, and aqueous) isolated from the inflorescence of *S. chilensis*. The inflorescence crude extract (ScIE) and fractions were administered by intraperitoneal route to mice at different doses. In an LPS-induced pleurisy model, inhibition of leukocyte influx was observed for the ScIE and all fractions tested, as compared to controls. Dichloromethane (ScDicF), butanolic (ScButF), and aqueous (ScAquF) were selected for further analysis as they showed the best inhibitory effects in leukocyte migration and inflammatory cytokine and chemokine production: TNF-*α*, CXCL1/KC, CXCL2/MIP-2, and CCL11/eotaxin-1. In LPS-stimulated J774A.1 cell line, ScIE and the ScDicF exhibited an inhibitory effect on nitric oxide (NO) production and downmodulated the COX-2 expression; ScAquF failed to modulate NO production and COX-2 expression. In phytochemical analysis, HPLC-UV-DAD chromatograms of ScDicF and ScAquF showed the main peaks with UV spectrum characteristics of flavonoids; chlorogenic acid and isoquercetin were the most present phytochemicals identified in the ScAquF, and a high number of n-alkanes was found in ScHexF. Our study was the first to address biological effects and correlate them to phytochemically characterized fractions from inflorescences of *S. chilensis*.

## 1. Introduction

*Solidago chilensis* Meyen (= *Solidago microglossa*) is a member of the Compositae family widely used in Brazilian folk medicine, as well as in other South American countries such as Chile, Argentina, Bolivia, and Paraguay. *S. chilensis*, popularly known as “Brazilian arnica,” can exert multiple biological effects and is used as an antidepressant, gastroprotective, diuretic, burn treatment, skin diseases, anticancer, antiedematogenic, and anti-inflammatory [1–9].

In addition to the popular uses, some reports have emphasized the anti-inflammatory activity of *S. chilensis*, where several types of extracts obtained, mainly from their rhizomes and leaves, were tested. In experimental models of inflammation, the modulation of these extracts in the inhibition of different inflammatory events, such as edema formation, mast cell degranulation, leukocyte recruitment, and inflammatory mediators' production (e.g., nitric oxide, interleukin-1*β*, and tumor necrosis factor-*α*), was observed [10–12]. These activities are related to the presence of flavonoid quercetrin, caffeoylquinic acid derivates, and flavonoid rutin [11, 12]; additionally, our recent investigation points to the presence of flavonoids derived from quercetin and kaempferol; and labdane diterpenes are partly responsible for these biological activities [13].

*S. chilensis* is part of the Brazilian National List of Medicinal Plants of Interest to Unified National Health System, and extracts obtained from rhizomes and inflorescences have been produced for anti-inflammatory use [13]. Although the activity of *S. chilensis* is evidenced in the literature, there are few studies with enriched/characterized fractions or isolated compounds, especially concerning extracts obtained from the inflorescences, relating them to the anti-inflammatory and antinociceptive activities already well described in this plant species [9, 14]. The generation of this knowledge can help in the identification of phytochemical markers that can be used in the future in the quality control of *S. chilensis*-based products.

In the present work, we evaluated, focusing on anti-inflammatory activity, the modulatory properties of different fractions obtained from the *S. chilensis* inflorescence crude extract. Based on the obtained results, we explored some mechanism of action of those fractions that presented greater biological activity and, in addition, we chemically characterized these fractions, in order to determine the main active compounds involved in the anti-inflammatory activity of *S. chilensis*.

## 2. Materials and Methods

### 2.1. Materials

Alamar Blue cell viability reagent was purchased from Invitrogen (Massachusetts, USA). Dexamethasone, diclofenac sodium, dimethyl sulfoxide (DMSO), Dulbecco's Modified Eagle Medium (DMEM), ethylenediaminetetraacetic acid (EDTA), fetal bovine serum (FBS), Laemmli sample buffer, lipopolysaccharide (LPS), phosphate-buffered saline (PBS), and protease inhibitor cocktail were purchased from Sigma-Aldrich (St. Louis, MO, USA). Ether, ethanol, ethyl acetate, dichloromethane, methanol, n-butanol, and n-hexane were purchased from Tedia Way (Fairfield, OH, USA). Nitrocellulose membranes were purchased from Amersham Pharmacia Biotech (San Francisco, CA, USA). The chromatographic solvents (acetonitrile, acetic acid, and trifluoroacetic acid) were purchased from Merck (Darmstadt, Germany). Monoclonal antibodies anti-COX-2, anti-goat IgG biotin-conjugated antibody, and streptavidin-conjugated horseradish peroxidase were purchased from Santa Cruz Biotechnology (Texas, USA). The CXCL2/MIP-2, CXCL1/KC, CCL11/eotaxin-1, and TNF-*α* enzyme-linked immunosorbent assay (ELISA) kit were purchased from R&D Systems (Minneapolis, MN, USA). The colorimetric kit used for the May-Grunwald-Giemsa method was purchased from Laborclin (Pinhais, PR, BR).

### 2.2. Plant Material

*Solidago chilensis* Meyen was cultivated at Phytomedicine Agroecological Platform (PAF), FIOCRUZ, Rio de Janeiro city, RJ, Brazil (22°93′52.74^″^S; 43°39′89.16^″^W). Their inflorescences were collected in the summer, between December and March, when they are produced due to increased sunlight. The central stems were collected from the same producers of inflorescence plants. The species identification was performed by Mariana Reis de Brito, Botany Herbarium of the Department of the Institute of Biology/UFRJ, and a voucher specimen was deposited under the code 32689/RFA. The study of the plant material was conducted under the register of Brazilian System for Management Genetic of Heritage and Associated Traditional Knowledge (SISGEN) (Process number AF96E16).

### 2.3. Ether-Ethanolic Crude Extract and Their Fraction Preparation

The inflorescences of the plant material supplied were oven-dried 35-40°C and pulverized in a knife mil. 200 grams of material was weighed and submitted to dynamic maceration with ether : ethanol (1 : 1-3 L) for 6 hours [15]. After this period, the ether : ethanol extract was filtered, evaporated under reduced pressure (in a rotary evaporator Büchi R-124®), lyophilized, and weighed (200 g; 4.94% yield). The material was referred to as the *Solidago chilensis* inflorescence crude extract (ScIE) and was stored at room temperature until use.

A part of the ScIE was separated for the pharmacological assays, and another portion was used to successive liquid : liquid partition with solvents of increasing polarity (n-hexane, dichloromethane, ethyl acetate, and n-butanol) to obtain the fractions. The ScIE was resuspended with water : methanol (4 : 1*v*/*v*, 500 mL), and then, the organic phase was evaporated. The aqueous portion was added with 500 mL of n-hexane and separated in a glass separatory funnel. This process was carried out five times, totaling the use of 2.5 L of solvent. The organic phase was evaporated, and a 0.46% yield of the hexane fraction (ScHexF) was obtained. Likewise, the remaining aqueous portion was added with 500 mL of dichloromethane, four times, totaling the use of 2 L of solvent. The organic phase was evaporated, and 0.42% yield of the dichloromethane fraction (ScDicF) was obtained. Always adding 500 mL of solvent to the aqueous portion at a time, the partition was carried out with the solvents ethyl acetate (ten times, using 5 L of solvent in total) and n-butanol (once), obtaining a yield of 1.08% for the acetalic fraction (ScAceF) and 2.26% for the butanolic fraction (ScButF). The remaining aqueous fraction (ScAquF) showed 2.84% yield.

### 2.4. Characterization and Identification of Ether-Ethanolic Crude Extract and Their Fractions

The high-performance liquid chromatography, coupled with ultraviolet spectroscopy, a diode-array detector (HPLC-UV-DAD) analysis, was carried out through two different methodologies, one developed to characterize flavonoids and phenolic compounds in *S. canadensis* [16] and another developed to characterize labdane diterpenes, such as solidagenone, commonly found in *S. chilensis* [15]. The first one was used in ScIE, ScDicF, and ScAquF. E the second one was used only in ScDicF.

The characterization through a HPLC-UV-DAD for flavonoids and phenolic compounds was performed with LiChrospher 100 RP-18 column (Merck™) for analytical scale with 250 mm length, 4 mm in diameter, and 5 *μ*m particle size, using C18 as stationary phase and DAD detector, type SPDM20A. A binary mixture was used as the eluent system, composed of acetic acid : water (1 : 40) and acetonitrile [16]. For the terpenes' investigation, a binary mixture with an isocratic eluent system, consisting of 0.05% trifluoroacetic acid and acetonitrile, was used [15]. Samples were diluted in the ratio of 10 mg/mL, and 10 *μ*L aliquots were injected. An analysis was performed at 25°C with a flow rate of 1.0 mL·min^−1^. The eluted compounds were analyzed for their absorption in UV at 310 nm for flavonoids and between 210 and 400 nm for terpenoids (solidagenone) [16, 17]. The analysis by HPLC-UV-DAD allowed the characterization of flavonoids and phenolic compounds through monitoring by internal normalization, coinjection of quercetin, and analysis of UV spectra, since all derivatives observed in the extract of *S. chilensis* have spectra in the UV characteristics of its aglycone (quercetin or kaempferol) in addition to chlorogenic acid, identified through its retention time in the methodology used, literature data, and UV spectrum, all also already rescribed for the genus *Solidago* [6, 16, 18–21]. The ScHexF was characterized through the Agilent 5973 GC-MS system (Agilent Technologies, USA). Helium was used as mobile phase and flow rate of 2 mL/min. The heating ramp followed the following schedule: 60°C (10 min); 60-120°C (60°C/min); 120-290°C (15°C/min); 290°C (17 min). The ionization voltage and temperature of injector and ion source were 70 eV and 260 and 300°C, respectively. The identification of organic compounds was performed using the MSD ChemStation E. 02.021431–Wiley 7N–1989-2011 software (Agilent Technologies, USA), which was used for data acquisition, library searching, and compound characterization.

### 2.5. Cell Culture

Murine macrophage cell line J774A.1 (ATCC TIB-67™) was maintained at Dulbecco's minimal essential medium (DMEM) with 10% FBS, and antibiotic mixture (penicillin, streptomycin, and ampicillin 100 units/mL), under predefined conditions of temperature at 37°C, 95% humidity, and 5% CO_2_.

### 2.6. Cytotoxicity Assay

The J774.A1 cell line was plated in a 96-well microplate (1 × 10^5^ cells/well, in quadruplicate) and cultured in the presence of ScIE and the five fractions at concentrations of 1, 10, 50, 100, and 200 *μ*g/mL for 48 hours (in 5% CO_2_, at 37°C). Cells cultured only with medium or culture medium containing 0.5% DMSO (extract diluent) were used as positive controls of cell viability. After 44 hours, 10% Alamar Blue was added to the wells. After 4 hours of incubation, the fluorescence signal was monitored in a microplate reader (Molecular Devices), Ex: 530-560 nm; Em: 590 nm [13].

### 2.7. Nitric Oxide Production

Macrophages J774.A1, incubated with ScIE and the five fractions or dexamethasone (20 pg/mL) for 1 hour, were plated in a 96-well microplate in a final concentration of 2.5 × 10^5^ cells/well, in quadruplicate (in 5% CO_2_ at 37°C). Macrophages were stimulated with LPS (1 *μ*g/mL), and after 24 hours, nitrite levels were determined in supernatants with Griess' reagent. Absorbance was read at 540 nm using a microplate reader (Molecular Devices). The concentration of nitrite was calculated from a sodium nitrite standard curve (range 6.5–100 *μ*M) [13].

### 2.8. Western Immunoblot Analysis

J774A.1 cells (1 × 10^6^ cells), pretreated with ScIE, hexane, dichloromethane, and aqueous fraction (10 *μ*g/mL) or dexamethasone (20 pg/mL) for 1 hour and stimulated with LPS (1 *μ*g/mL), were lysed in buffer (50 mM Tris, 150 mM NaCl, pH 8.0, 1% NP-40) containing protease inhibitor cocktail (1 : 1000). After 15 min of incubation on ice, nuclear proteins were collected (supernatant after centrifugation at 14.000 × g for 10 min at 4°C). The total protein content in the nuclear extracts was determined by the Lowry method (Bio-Rad, San Diego, CA, USA). The cell lysates were denatured in Laemmli sample buffer (1% sodium dodecyl sulfate (SDS); 5% 2-mercaptoethanol; 10% glycerol and 0.001% bromophenol blue) and heated at 95°C for 3 min. The samples (10 *μ*g of total protein) were resolved by 12% SDS-polyacrylamide gel electrophoresis (PAGE), and the proteins were transferred to nitrocellulose membranes. The membranes were blocked with Tween-PBS (0.5% Tween-20) containing 2% BSA and probed with the specific primary monoclonal antibodies anti-COX-2 (1 : 10000). After extensive washing in Tween-PBS, the nitrocellulose membranes were incubated with an anti-goat IgG biotin-conjugated antibody (1 : 20000) for 1 hour, followed by streptavidin-conjugated horseradish peroxidase (1 : 5000). Immunoreactive proteins were visualized by DAB staining. The bands were quantified by densitometry, using ImageJ (public domain) software programs [22–24].

### 2.9. Animals

Male Swiss Webster mice (20-25 g) were obtained from the Oswaldo Cruz Foundation Breeding Unit (Fiocruz, Rio de Janeiro, Brazil). Mice were kept in plastic cages with free access to food and fresh water in a room with controlled temperature (22 ± 2°C) and light (12 h/12 h light/dark cycle) at the experimental animal facility until use. All experimental procedures were performed according to The Committee on Ethical Use of Laboratory Animals of Oswaldo Cruz Foundation, under license P17/13-5.

### 2.10. Pleurisy

For induction of pleurisy, the animals received intrathoracic (i.t.) administration of 100 *μ*L of LPS (12.5 ng/animal) diluted in sterile PBS. Sensitized mice challenged with vehicle alone (saline) were used as the negative control group [25]. The animals were treated by intraperitoneal (i.p.) route, 1 hour before challenge with LPS, with ScIE at different doses (0.01, 0.1, 1, and 10 mg/kg), and in a second assay with ScIE and different fractions at a single dose of 10 mg/kg. In trials, the dexamethasone (5 mg/kg) and diclofenac sodium (50 mg/kg) groups were used as a positive control, and the group was not treated as a negative control. All treatments were given in the final volume of 200 *μ*L per animal by intraperitoneal (i.p.) route. For evaluation of pleurisy, the animals were euthanized 24 hours postchallenge and their chest cavities were washed with 1000 *μ*L PBS containing EDTA (10 *μ*M). The total leukocyte count was performed through a Neubauer chamber with dilution of the sample in Turk's dye, and differential leukocyte counting was performed using May-Grunwald-Giemsa-stained cytospins under bright-field microscopes (1000x magnification), and values are expressed as numbers of cells per cavity [22]. The washes were centrifuged (20,000 rpm-10 min), and the supernatant was collected and stored in a -70°C freezer for further analysis.

### 2.11. Determination of Proinflammatory Mediators

CXCL2/MIP-2, CXCL1/KC, CCL11/eotaxin-1, and TNF-*α* levels were measured in cell-free pleural washes recovered from mice 24 hours after LPS stimulation using a commercial ELISA kit purchased from R&D Systems (Minneapolis, MN, USA). The results are expressed as ng/mL based on a standard curve [22].

### 2.12. Statistical Analysis

Results are reported as the mean ± standard error of the mean (SEM) and were statistically analyzed by means of analysis of variance (ANOVA) followed by the Newman-Keuls-Student test or Student's *t*-test. Values of *p* ≤ 0.05 were regarded as significant.

## 3. Results

### 3.1. Effect of S. chilensis Inflorescence Crude Extract on LPS-Induced Pleurisy

**Figure 1:**
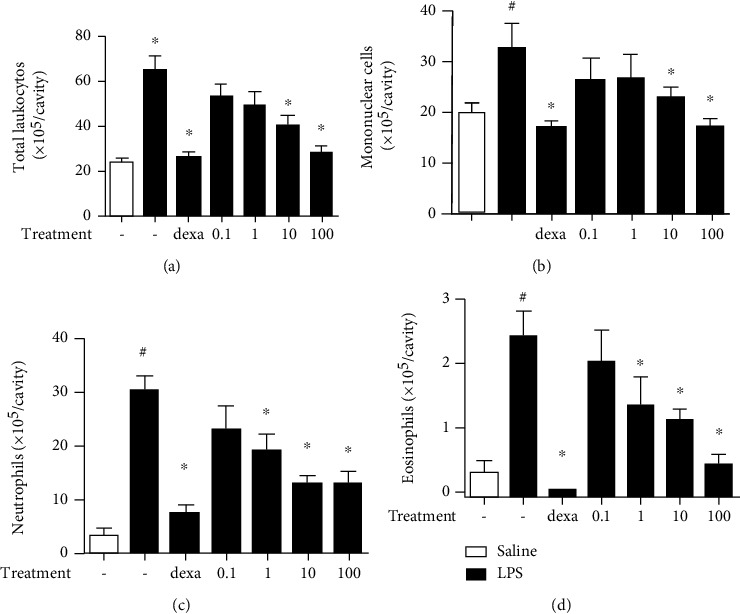
Effects of *S. chilensis* crude extract pretreatment (0.1, 1, 10, and 100 mg/kg) on pleural accumulation of total leukocytes (a), mononuclear cells (b), neutrophil (c), and eosinophil (d) populations in the pleural cavity of mice (*n* = 6) challenged with LPS (12.5 ng/animal). Dexamethasone was used as the reference drug. Mice were treated 1 hour before the challenge. Data represent mean ± standard error of the mean (SEM) (*n* = 6). Representative of two independent experiments performed. ^#^*p* < 0.05 stimulated group (LPS) vs. nonstimulated group (saline); ^∗^*p* < 0.05 treated vs. untreated group.

To verify the anti-inflammatory activity of the *S. chilensis* inflorescence crude extract (ScIE), mice were previously treated with dexamethasone (5 mg/kg, by intraperitoneal (i.p.) route) or with different doses of the ScIE (0.1, 1, 10, and 100 mg/kg, i.p.), and after 1 hour challenged with LPS (12.5 ng/cavity, i.t.). After 24 hours, the pleural inflammation induced by LPS is characterized by an intense leukocyte accumulation, with a marked increase in neutrophil and eosinophil numbers in the untreated group (LPS) when compared to the untreated and nonstimulated group (saline), as observed in Figure 1. Previous treatment with dexamethasone reduced basal levels of the leukocyte infiltrate including the polymorphonuclear cells (Figure 1). As for the ScIE, from the 10 mg/kg dose, a significant reduction in the total number of leukocytes was observed (Figure 1(a)). Mononuclear cell influx is inhibited by doses of 10 and 100 mg/kg of the extract (Figure 1(b)), and in the neutrophil and eosinophil populations, doses from 1 to 100 mg/kg of the extract were effective in reducing the migration, compared to the untreated group (Figures 1(c) and 1(d)). As the results between the 10 and 100 mg/kg doses of ScIE were not discrepant in all parameters, the 10 mg/kg dose was chosen for the assay with the *S. chilensis* fractions.

### 3.2. Effect of S. chilensis Fractions on LPS-Induced Pleurisy

A second pleurisy trial was performed to determine whether there would be differentiated anti-inflammatory activity between the *S. chilensis* fractions at the predetermined dose of 10 mg/kg. Swiss Webster mice, previously treated with dexamethasone, diclofenac sodium, ScIE, and the different fractions obtained from *S. chilensis* (10 mg/kg), were stimulated with LPS (i.t.) after 1 h.

**Figure 2:**
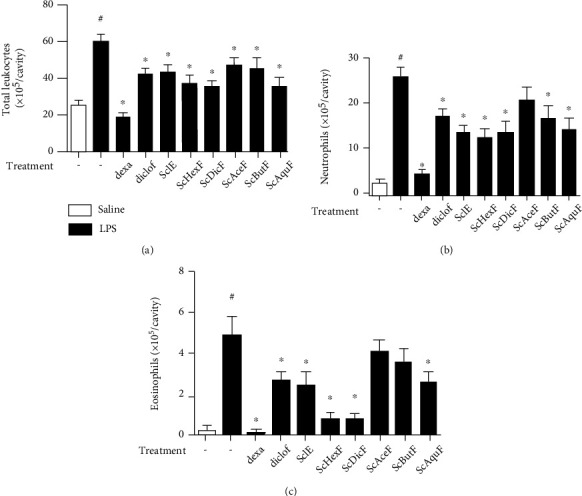
*In vivo* effect of the crude extract and different fractions of *S. chilensis* (10 mg/kg) on the recruitment of total leukocytes (a) and neutrophil (b) and eosinophil (c) populations in the pleural cavity of Swiss Webster mice stimulated (i.t.) with LPS (12.5 ng/per cavity). Dexamethasone (5 mg/kg, i.p.) and diclofenac sodium (10 mg/kg) were used as reference drugs. Mice were treated 1 hour before the challenge. Data represent mean ± standard error of the mean (SEM) (*n* = 6). Representative of three independent experiments performed. ^#^*p* < 0.05 stimulated group (LPS) vs. nonstimulated group (saline); ^∗^*p* < 0.05 treated vs. untreated group.

Similar to the reference drug-treated groups, both the ScIE and the fractions significantly reduced the influx of total leukocytes at the site of the induced inflammation (Figure 2(a)). By analyzing neutrophil recruitment, the reference drug, dexamethasone, reduced almost to baseline levels when compared to the untreated group (Figure 2(b)). Diclofenac sodium, as well as the *S. chilensis* butanolic fraction (ScButF), presented approximately 19% reduction. The ScIE and the dichloromethane (ScDicF) and aqueous fractions (ScAquF) reduced the recruitment by around 24%, while the hexane fraction (ScHexF) presented better results with 28% inhibition (Figure 2(b)). There was no significant reduction for the group treated with the acetalic fraction (ScAceF) (Figure 2(b)).

The ScIE and ScAquF reduced by approximately 52% eosinophil accumulation, while the ScHexF and ScDicF showed a better inhibition profile with a 79% of reduction. On the other hand, ScAceF and ScButF, although presenting a tendency, did not obtain statistically significant differences from the LPS group (Figure 2(c)). Given the results, ScHexF, ScDicF, and ScAquF were selected to continue the studies.

### 3.3. Effect of S. chilensis Fractions on the Production of Chemotactic Inflammatory Mediators

LPS stimulus induces the production of several inflammatory mediators, including cytokines and chemokines [25, 26]. To assess the difference in reducing the *in vivo* migration of leukocytes by the different *S. chilensis* fractions, we evaluated the production of TNF-*α*, CXCL1/KC, CXCL2/MIP-2, and CCL11/eotaxin-1 in pleural washes after the LPS (i.t.) challenge.

**Figure 3:**
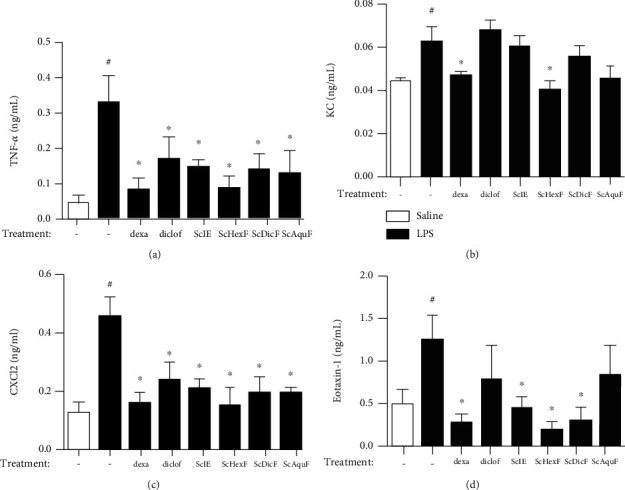
*In vivo* effect of crude extract and different fractions of *S. chilensis* (10 mg/kg) on the production of TNF-*α* (a), CXCL1/KC (b), CXCL2/MIP-2 (c), and CCL11/eotaxin-1 (d) mediators in the pleural lavage of Swiss Webster mice (*n* = 6) challenged with LPS (12.5 ng/animal, i.t.). Dexamethasone (5 mg/kg, i.p.) and diclofenac sodium (10 mg/kg, i.p.) were used as reference drugs. Data represent mean ± standard error of the mean (SEM) (*n* = 6). One representative ELISA from two independent experiments performed. ^#^*p* < 0.05 stimulated group (LPS) vs. nonstimulated group (saline); ^∗^*p* < 0.05 treated vs. untreated stimulated group (LPS).

After 24 hours of LPS stimulation, a significant increase in the inflammatory mediator's production in the untreated was observed and stimulated with the LPS group when compared to the untreated and unstimulated group (saline) (Figure 3). In relation to the production of TNF-*α* mediator, a reduction was observed in all the treated groups when compared to the untreated LPS group. The dexamethasone and ScHexF groups had the best inhibition profiles, at approximately 89% and 86%, respectively (Figure 3(a)). When CXCL1/KC chemokine levels were determined, only dexamethasone and ScHexF were able to significantly attenuate this cytokine production (Figure 3(b)). CXCL2/MIP-2 level determination demonstrated that all treated groups showed inhibition of this chemokine release (Figure 3(c)). The inhibition of CXCL2/MIP-2 production is in line with the neutrophil recruitment reduction observed in Figure 2(b). As shown in Figure 3(d), there was a reduction of CCL11/eotaxin-1 production in the dexamethasone-treated group. The ScIE and the ScDicF had a similar inhibitory profile, while the ScHexF showed a more pronounced reduction (91% of inhibition) (Figure 3(d)). These results corroborate with the inhibition of eosinophil recruitment observed in Figure 2(c). The groups treated with diclofenac or ScAquF did not inhibit CCL11/eotaxin-1 production (Figure 3d). It is noteworthy that the ScHexF was the only *S. chilensis* fraction that reduce all the cytokine/chemokine production analyzed by this study (Figure 3).

### 3.4. Cytotoxic Effect of the S. chilensis Crude Extract and Fractions in Cell Line J774A.1

In order to verify whether the ScIE or their fractions have any cytotoxic effect that would impair the cell population used throughout the *in vitro* study, a cell viability analysis was performed with the J744A.1 murine macrophage cell line. As observed in the ScIE, most fractions were cytotoxic to almost 100% of the cell population at concentrations of 100 and 200 *μ*g/mL (Table 1). The exception was for the ScAquF, which even at the highest concentrations maintained around 80% of the viable cell population. The ScDicF and ScAceF at the concentrations of 50 *μ*g/mL were cytotoxic to 37.5% and 70.4% of the population, respectively. Because of these results, the concentration of 10 *μ*g/mL was chosen for the following experiments.

### 3.5. Evaluation of the Inhibitory Capacity of S. chilensis Fractions in the NO Production and COX-2 Expression In Vitro

**Figure 4:**
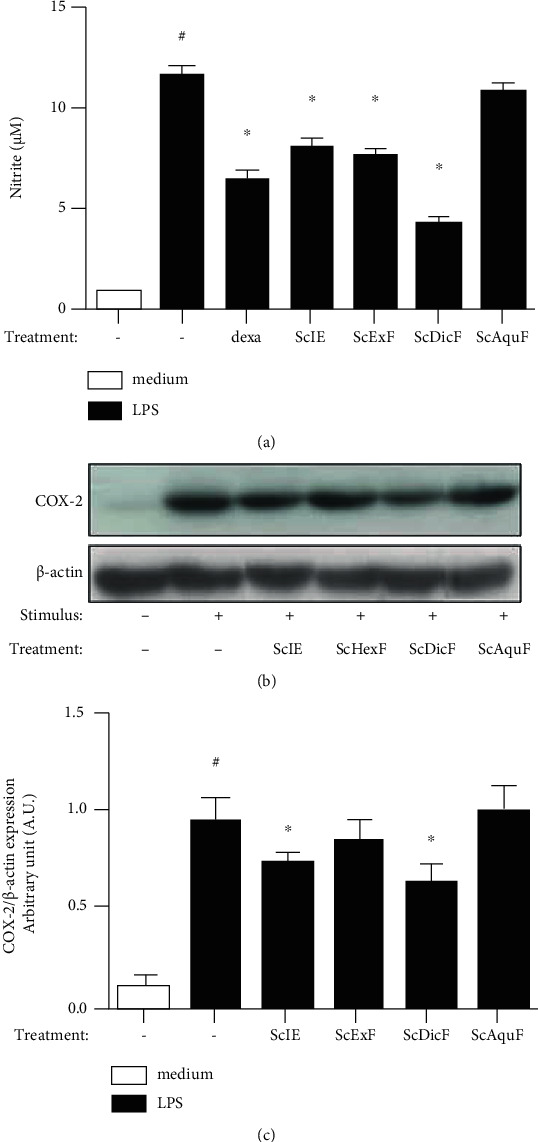
(a) The NO production was determined by the Griess reagent through the supernatant collected 24 h after the LPS simulation in treated cells with *S. chilensis* fractions (10 *μ*g/mL). Dexamethasone (20 pg/mL) was used as the reference drug. (b, c) Effect of *S. chilensis* fractions in COX-2 expression in LPS-induced J774A.1 cells. Data represent mean ± standard error of the mean (SEM) (*n* = 4). Representative of two independent experiments performed. ^#^*p* < 0.05 stimulated group (LPS) vs. nonstimulated group (medium); ^∗^*p* < 0.05 treated vs. untreated stimulated group (LPS).

We previously demonstrated that the 10 *μ*g/mL concentration of the ScIE significantly reduced nitric oxide (NO) production in the LPS-stimulated J774.A1 cell line [13]. Therefore, we indirectly quantify the NO production in LPS-stimulated J744A.1 cells previously treated with ScHexF, ScDicF, and ScAquF (10 *μ*g/mL) (Figure 4(a)). After 24 hours of stimulation, there was a significant increase in the production of the stable metabolite of NO (nitrite) in the LPS-stimulated cells when compared to the untreated and unstimulated group (medium) (Figure 4(a)). Dexamethasone, ScIE, and ScHexF presented a percentage of approximately 30% of inhibition of the NO mediator production, while the SCDicF obtained the best reduction profile with 59% of inhibitory capacity. However, the ScAquF did not show any inhibitory activity in J744A.1 cell NO production (Figure 4(a)). In addition, ScIE and ScDicF inhibited the COX-2 expression in stimulated cells (Figures 4(b) and 4(c)). Differently, ScHexF and ScAquF failed to inhibit COX-2 expression.

### 3.6. Phytochemical Constituents of the S. chilensis Crude Extract and Fractions

Based on the findings of biological activity presented by the fractions, we performed a phytoconstituent investigation in ScIE and their fractions (ScDicF, ScHexF, and ScAquF). Our group previously showed that ScIE contains 13.8% of flavonoids derived from quercetin and kaempferol, and 23.1% of diterpenes, probably furan labdanes [13].

**Figure 5:**
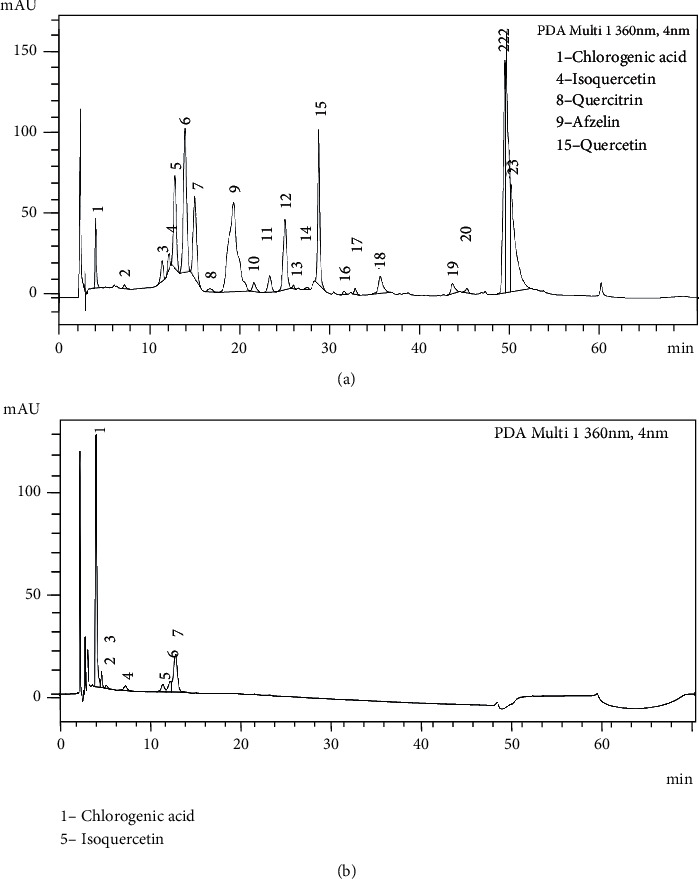
HPLC-UV-DAD of the ScDicF (a) and ScAquF (b) for flavonoid characterization. The chromatogram A showed the main peaks with UV spectrum characteristics of chlorogenic acid (1), isoquercetin (4), quercitrin (8), afzelin (1), and quercetin (15). And the chromatogram B showed the main peaks with UV spectrum characteristics of chlorogenic acid (1) and isoquercetin (5). Chromatographic conditions: temperature = 250°C; flow rate = 1.0 mL min^−1^; *ʎ* = 360 nm; Inj.V. = 10 *μ*L. Mobile phase : acetonitrile (eluent A) and acetic acid : water (1 : 40) (eluent B). In the first 15 min, 14% of the A eluent and 84% of the B eluent were used. 35% of the eluent A in the following 30 min and 100% of eluent A in the last 2 minutes.

The HPLC-UV-DAD chromatograms of ScDicF and ScAquF (Figure 5) showed the main peaks with UV spectrum characteristics of flavonoids. ScDicF contains afzelin (17%), quercetin (7.2%), chlorogenic acid (2.1%), isoquercetin (0.8%), quercitrin (0.4%), and an unknown flavonoid (10.4%) at 11.98 min retention time (Figure 5(a)). The ScAquF contains chlorogenic acid (58.2%), isoquercetin (4.1%), and an unknown flavonoid (25.1%) at 12.76 min retention time (Figure 5(b)). The flavonoid identification was carried out by comparison of the reference flavonoids, as quercetin coinjection, and by comparing retention time in the methodology used (UV spectrum) and literature data.

**Figure 6:**
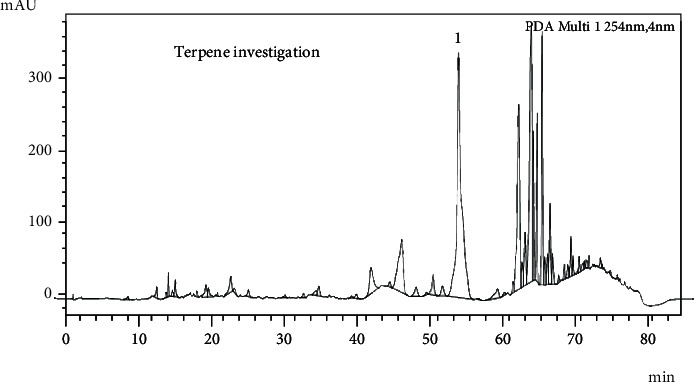
HPLC-UV-DAD of the ScDicF for the characterization of terpenes. The presence of the labdanic diterpene solidagenone was not found (retention time 71-72 min), but a great diversity of signs suggestive of terpenes can be observed, as well as a great sign (1) of a substance still not identified (26% of the area, retention time = 53.71 min). Chromatographic conditions: temperature = 300°C; flow rate = 1.0 mL min^−1^; *ʎ* = 254 nm; Inj.V. = 10 *μ*L. Isocratic elution system; 0.05% trifluoroacetic acid (54.94 mL) and acetonitrile (27.06 mL).

Through the HPLC-UV-DAD method, the presence of the labdanic diterpene solidagenone was not found in ScDicF, which has a retention time between 71 and 72 min (in the absorbance range of 254 nm). However, we observed a great diversity of signs suggestive of terpenes, as well as a great sign of a substance still not identified by us with 26% of the area, in the retention time of 53.71 min (Figure 6). This substance will be isolated for further identification.

The GC-MS chromatogram analysis showed a high number (35.4%) of n-alkanes (paraffins) in the ScHexF; in addition, the labdanic diterpene (solidagenone; 2.2%), ex bornyl acetate (0.5%), guaiene (0.42%), and Stigmasta-7,25-dien-3*β*-ol (0.58%) were also identified.

## 4. Discussion

*Solidago chilensis* is a plant species used in Brazilian popular medicine for the treatment, mainly of inflammatory pathologies, but there is little scientific evidence associating its therapeutic effect with the present metabolites [15, 27]. Although the literature has already described effective doses for the anti-inflammatory activity of *S. chilensis* extracts in animal experimentation models, these studies were based on extractions made from rhizomes or aerial parts, or from the aqueous extract of inflorescences [10–12], which differs from the proposal of this work, where only inflorescences were used in extraction with solvents of different polarities.

Among the doses tested in our study, the dose of 10 mg/kg was chosen because it was efficient in the proposed model, corroborating the results found by Goulart et al. [10]. Although all fractions, as well as ScIE, modulated the total leukocyte infiltrate, the ScAceF and ScButF were not able to significantly inhibit the migration of polymorphonuclear cells. The fact that some fractions do not have the same pharmacological effect as ScIE can be explained by the fact that the phytocomponents responsible for the effects found in the ScIE were separated according to the affinity of secondary metabolites to from the solvents used (due to the polarity), or by the loss of the synergistic effect between these compounds during the liquid-liquid partition [28].

TNF-*α* is responsible for endothelial cell activation increasing the expression of adhesion molecules in the vascular endothelium, allowing leukocyte transmigration to the inflammation site. This cytokine is a potent neutrophil activator, mediating chemotaxis, adherence, degranulation, and respiratory burst, being produced not only by lymphocytes, macrophages, and active neutrophils but also by vascular endothelial, natural killer, and mast cells [29]. The data presented in this work show that the ScIE and the three tested fractions of *S. chilensis* significantly reduced the TNF-*α* values. It has already been seen that *S. chilensis* can modulate the levels of TNF-*α*, as well as the inhibition of the cytokine IL-1*β* in mouse experimentation models, with extracts from the rhizomes [10, 11]. It is suggested that the downmodulation of TNF-*α* and IL-1*β* attributes an anti-inflammatory mechanism via inhibition of the activation of adhesion molecules, and blocking the release of specific cytokines in cell recruitment, such as chemokines of the C-X-C family [10, 11, 30].

Neutrophils are essential effector cells of the immune system and are considered the first line of defense against infections from fungi and bacteria. These cells, however, can contribute to tissue damage in diseases of acute processes, such as acute lung injury, and in the acute phase of chronic diseases such as rheumatoid arthritis. The destructive potential of neutrophils depends directly on the control of their recruitment to tissues [31]. The chemokines KC and MIP-2 are potent chemoattractants, and activators of neutrophils are known to have a crucial role in the migration of these cells from blood vessels to the sites of inflammation [32].

According to Filippo et al. [33], the KC and MIP-2 chemokines are produced by the activation of Toll-like receptors (TLRs), mainly in macrophages residing in the tissue. Both proteins are produced via MyD88, but MIP-2 is also synthesized via TRIF. In this work, a reduction of MIP-2 in ScIE and all tested fractions was observed, when compared to the group that did not receive treatment; however, the ScHexF was the only one that modulated KC production. The ScHexF may be the only one capable of interfering in the recruitment of neutrophils via the MyD88 pathway. Once maximum neutrophil infiltration occurs when both chemokines are concomitantly expressed, and inhibition of both at the same time prevents leukocyte recruitment [33]. Thus, the ScHexF would have the best antichemotactic effect of neutrophils, among all the fractions studied.

Eosinophils are leukocytes of multiple functionalities, acting in the pathogenesis of innumerable inflammatory processes, being more prominent in allergic processes and helminth infections [34]. Among the many molecules involved in the chemotaxis of this cell population, eotaxin-1, secreted mainly by cells of the bronchial epithelium, endothelial cells, and the eosinophil itself, acts as a potent chemoattractant, being just enough for local eosinophilic recruitment [35, 36]. The inhibition of eosinophilic populations in the washings of animals treated with some specific fractions (ScIE and ScHexF, ScDicF and ScAquF, the latter in lesser quantities) was an unprecedented fact of this study since the experimental models adopted in other studies involved the stimulation of the inflammatory process via carrageenan, a substance that generates inflammatory polymorphonuclear infiltrates with the majority presence of neutrophils [10–12]. Therefore, these groups were assessed for modulation of eotaxin-1 levels. Modulation of eotaxin-1 was possible in groups of mice previously treated with ScIE and the ScHexF and ScDicF, indicating that *S. chilensis* may have antiallergic activity, which has not been previously studied, but which is not uncommon in the Compositae family [27, 37]. Although the ScAquF was effective in modulating the migration of eosinophils, the levels of eotaxin-1 did not decrease significantly; therefore, it is necessary to investigate other modulation mediators and pathways, such as prostaglandins and leukotriene.

Our group evaluated the safety of this extract and fractions *in vitro*, and except for ScAquF, which maintained a cell viability profile greater than 80% in all concentrations tested, the other fractions, as well as the ScIE, showed high toxicity at doses of 100 and 200 *μ*g/mL, with almost 100% of cell death. The ScDicF was the only one that at a concentration of 50 *μ*g/mL made more than half of the cell population unviable. In order to use cells previously treated with the *S. chilensis* fractions, which maintained a cell viability profile equal to or greater than 70%, the concentration of 10 *μ*g/mL was chosen as the maximum working concentration. The results obtained in this work can be associated with the findings of Barros et al. [2], where the concentration of 10 *μ*g/mL of the methanolic extract of the leaves of *S. chilensis* did not show a cytotoxic effect in an L929 murine fibroblast strain [2].

NO is an important molecule in the defense system, acting as a vasodilator and cytotoxic agent mediated by macrophages in infectious processes [38, 39]. Although the literature has already described the ability of *S. chilensis* to modulate NO [10, 11], our group analyzed the modulation *in vitro* using a murine macrophage cell lineage treated with ScIE and different fractions obtained from inflorescences. Like the *in vivo* assay, a dose-response curve was constructed with ScIE. The data presented showed that only the ScAquF failed to inhibit the production of NO.

COX-2 is an enzyme produced by resident cells involved in the inflammatory process (macrophages, mast cells, and others) and is responsible for the synthesis of prostaglandins and thromboxanes, through stimulation with IL-1*β*, TNF-*α*, endotoxins, and others [40]. We observed for the first time that components present in the ScIE and dichloromethane fraction of *S. chilensis* have the property of significantly modulating the expression of COX-2. The reduction of COX-2 expression leads to the belief that there is some inhibitory activity via the gene pathway, where the protein is not being synthesized [41]. However, that does not rule out the fact that ScIE and fractions, even ScHexF or ScAquF that did not show an indication of reduced expression, cannot be inhibiting the functionality of the enzyme, as occurs in the case of nonsteroidal anti-inflammatory drugs, such as aspirin, or some selective for COX-2 such as celecoxib [40]. This evidence requires new tests to obtain more conclusive results, but given all the data presented, it can be suggested that a large part of the pharmacological activity is found in phytocomponents present in the dichloromethane fraction, with the main effect may be the inhibitory expression of COX-2 at the molecular level.

To identify the compounds that could be involved in the pharmacological effects found in this work, the ScIE, hexane, dichloromethane, and aqueous fractions were subjected to chromatographic analyses for the investigation of phytochemicals. The analysis by HPLC-UV-DAD showed that both the ScIE and the dichloromethane fraction are constituted by a large number of flavonoids derived from quercetin and kaempferol, between aglycone and glycosides, since the absorbance observed in UV is characteristic only of the aglycone. As for the aqueous fraction, in the same methodology, the presence of the isoquercitrin flavonoid and a large amount of chlorogenic acid were identified.

Although the majority of compounds have not been fully identified, most have quercetin as a flavonoid skeleton with strong antioxidant activity represented there, and already well described in the literature [42]. This antioxidant capacity of the flavonoids could explain the modulation of NO levels found in ScIE, and the ScDicF. Barros et al. [2] demonstrated the antioxidant potential of quercitrin and afzelin isolated from *S. chilensis* in a DPPH trial, as well as the ability of flavonoid quercitrin gallate (derived from quercetin) to modulate NO levels through inhibition of production, at the level of gene transcription, of the enzyme responsible for its synthesis (iNOS) in a lineage of RAW 264.7 macrophages [43].

Rao-Manjeet and Ghosh [44] demonstrated the potential of quercetin in modulating the levels of TNF-*α* in a lineage of RAW 264.7 macrophages, going according to the findings of this study in the ScIE and the dichloromethane fraction. Quercetin is already described for having anti-inflammatory activity through the reduction of leukotriene B4, prostaglandin E2 (PGE2), chemokines KC, MIP-2, RANTES, and CXCL8, all with chemoattractive properties of leukocytes [42, 45, 46]. Part of this activity is attributed to the ability of quercetin to inhibit the formation of the NF-*κ*B transcription complex and to interrupt signaling between TLR4 and MyD88 [47]. Chlorogenic acid, found in large amounts in the aqueous fraction, also has anti-inflammatory activity attributed to the ability to suppress the production of NO, iNOS, and PGE2, also by blocking NF-*κ*B [48, 49].

The labagenic diterpene solidagenone has been described as the main constituent of rhizomes of *S. chilensis*, and it has already been attributed some pharmacological effects such as antiulcer, gastroprotective, and immunomodulator [15, 50]. Using the methodology validated by Valverde et al. [15] for the identification, mainly, of ketone or lactonic groups through HPLC-UV-DAD, the presence of solidagenone in the ScIE and the ScDicF was investigated. Our results converge with those of the literature, where the ether-ethanolic extract of the inflorescences presented a considerable amount of solidagenone [15]. On the other hand, the ScDicF analysis was negative for the presence of solidagenone.

In the hexane fraction, in addition to a large amount of grease found, characteristics of the waxes found on the surfaces of flowers, the presence of solidagenone, exabornyl acetate, and guaiene were identified by GC-MS. The literature describes little about the pharmacological activities of these compounds. Bornyl acetate has been described as having analgesic and anti-inflammatory activity [51], but it is not guaranteed that the low percentage of this substance in the studied fraction is responsible for the pharmacological activity observed in this work. The same is true with solidagenone.

According to our analysis, quercetin and its derivatives would be excellent biomarkers of anti-inflammatory activity for extracts from the inflorescences of *Solidago chilensis*. It was found in the ScIE and the ScDicF in good proportions, and its anti-inflammatory and antioxidant properties are well described in the literature [42]. Although the ScHexF has shown good results in immunopharmacological tests, new analyses for the investigation of phytochemicals must be performed to improve the understanding of its effects. Solidagenone, despite being found in good proportions in the ScIE, needs further studies in more detail and in an isolated way about its pharmacological activities, as it is a metabolite little investigated in the literature, being a possible target of future studies.

## 5. Conclusion

The inflorescence crude extract and different fractions obtained from the ether-ethanolic extract of *S. chilensis* showed a differentiated anti-inflammatory effect in the proposed methodologies for evaluating *in vivo* and *in vitro* parameters. After phytochemical investigation, it is concluded that quercetin (and its derivates) could be used as biomarkers for quality control of the anti-inflammatory activity of this species. More studies should be carried out to improve the understanding of the mechanism of action of the anti-inflammatory property of *S. chilensis*, as well as to confirm, and further explore, a possible antiallergic effect seen in this work, which had not been reported in the literature.

## Figures and Tables

**Table 1:** Cytotoxic effect of crude extract and hexane, dichloromethane, acetalic, butanolic, and aqueous fractions from *S. chilensis* in murine macrophages J774A.1 (1 × 10^5^ cells/well; *n* = 4, one representative assay of two independent experiments) expressed as a percentage of viable cells (%).

Sample	Dose (*μ*g/mL)	Viability (%)
Control	—	100

DMSO	—	100

SclE	1	100
	10	100
	50	90.7
	100	6.8
	200	0

ScHexF	1	100
	10	100
	50	88.9
	100	0.6
	200	0

ScDicF	1	82
	10	85.6
	50	37.5
	100	0
	200	0

ScAceF	1	83.9
	10	88.1
	50	70.4
	100	6.9
	200	0

ScButF	1	100
	10	100
	50	85.1
	100	0
	200	0

ScAquF	1	83.4
	10	83.6
	50	88.9
	100	81.6
	200	83.5

## Data Availability

The datasets generated and/or analyzed during the present study are available from the corresponding authors upon reasonable request.

## References

[1] Locateli G., de Oliveira Alves B., Miorando D. (2020). Antidepressant-like effects of solidagenone on mice with bacterial lipopolysaccharide (LPS)-induced depression. *Behavioural Brain Research*.

[2] de Barros M., Mota da Silva L., Boeing T. (2016). Pharmacological reports about gastroprotective effects of methanolic extract from leaves of Solidago chilensis (Brazilian arnica) and its components quercitrin and afzelin in rodents. *Naunyn Schmiedebergs Arch Pharmacology*.

[3] Pio Corrêa M. (1984). “Dicionário de plantas úteis do Brasil e das exóticas cultivadas”, Rio de Janeiro: Ministério da Agricultura. *IBDF*.

[4] Mors W., Rizzini C., Pereira N. (2000). *Medicinal plants of Brazil*.

[5] Facury Neto M., Fagundes D., Beletti M., Novo N., Juliano Y., Penha-silva N. (2004). Systematic use of Solidago microglossa dc in the cicatrization of open cutaneous wounds in rats. *Brazilian Journal of Morphological Sciences*.

[6] Lorenzi H., Matos F. (2008). *Plantas medicinais no brasil: nativas e exóticas cultivadas*.

[7] Catarino H., de Godoy N., Scharlack N. (2015). Ingap 670-nm laser therapy combined with a hydroalcoholic extract of Solidago chilensis meyen in burn injuries. *Lasers in Medical Science*.

[8] Malpezzi-marinho E., Molska G., Freire L. (2019). Effects of hydroalcoholic extract of Solidago chilensis meyen on nociception and hypernociception in rodents. *BMC Complementary and Alternative Medicine*.

[9] Valverde S., Santos B., de Oliveira T., Gonçalves G., de Sousa O. (2021). Solidagenone from Solidago chilensis meyen inhibits skin inflammation in experimental models. *Basic & Clinical Pharmacology & Toxicology*.

[10] Goulart S., Moritz M. I. G., Lang K. L., Liz R., Schenkel E. P., Fröde T. S. (2007). Anti-inflammatory evaluation of _Solidago chilensis_ Meyen in a murine model of pleurisy. *Journal of Ethnopharmacology*.

[11] Liz R., Vigil S., Goulart S., Moritz M., Schenkel E., Frode T. (2008). The anti-inflammatory modulatory role of Solidago chilensis meyen in the murine model of the air pouch. *Journal of Pharmacy and Pharmacology*.

[12] Tamura E., Jimenez R., Waismam K., Gobbo-Neto L. (2009). Inhibitory effects of _Solidago chilensis_ Meyen hydroalcoholic extract on acute inflammation. *Journal of Ethnopharmacology*.

[13] de Brito T., Amendoeira F., de Oliveira T., Frutuoso V., Ferraris F., Valverde S. (2020). Extract of Solidago chilensis meyen inflorescences: cytotoxicity and inhibitory activity on nitric oxide synthesis in activated macrophage cell line J774A.1. *Brazilian Journal of Pharmaceutical Sciences*.

[14] Valverde S., Souza S., de Oliveira T. (2020). Chemical composition and antinociceptive activity of volatile fractions of the aerial parts of Solidago chilensis (compositae). *Rodriguésia*.

[15] Valverde S., Azevedo R., Tomassini T. (2009). Utilização de clae como paradigma na obtenção e controle do diterpeno solidagenona a partir de inflorescências de Solidago chilensis meyen (arnica brasileira). *Revista Brasileira de Farmacognosia*.

[16] Apáti P., Szentmihályi K., Balázs A. (2002). HPLC analysis of the flavonoids in pharmaceutical preparations from Canadian goldenrod (Solidago canadensis). *Chromatographia Supplement*.

[17] de Oliveira T., Bastos B., Kelly A., Monteiro S., Valverde S. (2017). Flavonoids characterization using HPLC-UV-PDA in tincture produced from inflorescences of cultivated Solidago chilensis Meyen in Itaipava (RJ). *Revista Fitos*.

[18] Torres L., Akisue K., Roque F. (1987). Quercitrina em Solidago microglossa DC, A Arnica do Brasil. *Revista Farmácia e Bioquímica da USP*.

[19] Vila R., Mundina M., Tomi F. (2002). Composition and antifungal activity of the essential oil of Solidago chilensis. *Planta Medica*.

[20] Apáti P. (2003). *Antioxidant constituents in Solidago canadensis L. and its traditional phytopharmaceuticals [Ph.D. thesis]*.

[21] Jiang T., Huang B., Qin P. (2006). A survey of chemical and pharmacological studies on Solidago. *Journal of Chinese Integrative Medicine*.

[22] Ferraris F., Moret K., Figueiredo A., Penido C., Henriques M. (2012). Gedunin, a natural tetranortriterpenoid, modulates t lymphocyte responses and ameliorates allergic inflammation. *International Immunopharmacology*.

[23] Conte F., Ferraris F., Costa T. (2015). Effect of gedunin on acute articular inflammation and hypernociception in mice. *Molecules*.

[24] Pádua T., Torres N., Candéa A. (2018). Therapeutic effect of lipoxin A4 in malaria-induced acute lung injury. *Journal of Leukocyte Biology*.

[25] Penido C., Castro-faria-neto H., Vieira-de-abreu A. (2001). LPS induces eosinophil migration via CCR3 signaling through a mechanism independent of rantes and eotaxin. *American Journal of Respiratory Cell and Molecular Biology*.

[26] Penido C., Vieira-de-abreu A., Bozza M., Castro-faria-neto H., Bozza P. (2003). Role of monocyte chemotactic protein-1/CC chemokine ligand 2 on gamma delta T lymphocyte trafficking during inflammation induced by lipopolysaccharide or Mycobacterium bovis bacille Calmette-Guérin. *Journal of Immunology*.

[27] de Oliveira T., Silva F., Kelly A., Valverde S. (2017). Solidago medicinais. *Revista Brasileira de Plantas Medicinais*.

[28] Queiroz S., Collins C., Jardim I. (2001). Métodos de extração e/ou concentração de compostos encontrados em fluidos biológicos para posterior determinação cromatográfica. *Química Nova*.

[29] Borish L., Stenki J. (2003). 2\. Cytokines and chemokines. *The Journal of Allergy and Clinical Immunology*.

[30] Ruth J., Amin M., Woods J. (2005). Accelerated development of arthritis in mice lacking endothelial selectins. *Arthritis Research & Therapy*.

[31] Sadik C., Kim N., Luster A. (2011). Neutrophils cascading their way to inflammation. *Trends in Immunology*.

[32] Day R., Link D. (2012). Regulation of neutrophil trafficking from the bone marrow. *cellular and Molecular Life Sciences*.

[33] Filippo K., Henderson R., Laschinger M., Hogg N. (2017). Neutrophil chemokines KC and macrophage-inflammatory protein-2 are newly synthesized by tissue macrophages using distinct TLR signaling pathways. *The Journal of Immunology*.

[34] Rothenberg M., Hogan S. (2006). The eosinophil. *Annual Review Immunology*.

[35] Wu D., Zhou J., Bi H. (2014). CCL11 as a potential diagnostic marker for asthma?. *Journal of Asthma*.

[36] Palomino D., Marti L. (2015). Chemokines and immunity. *Einstein (são paulo)*.

[37] Chandrashekhar V., Halagali K., Nidavani R. (2011). Anti-allergic activity of German chamomile (_Matricaria recutita_ L.) in mast cell mediated allergy model. *Journal of Ethnopharmacology*.

[38] Cerqueira N., Yoshida W. (2002). Óxido nítrico: revisão. *Acta Cirúrgica Brasileira*.

[39] Jo H., Kim Y., Nam S. (2008). The inhibitory effect of quercitrin gallate on iNOS expression induced by lipopolysaccharide in balb/c mice. *Journal of Veterinary Science*.

[40] Carvalho W., Carvalho R., Santos F. (2004). Analgésicos inibidores específicos da ciclooxigenase-2: avanços terapêuticos. *Revista Brasileira de Anestesiologia*.

[41] Yo Y., Li X., Qu L. (2016). DXXK exerts anti-inflammatory effects by inhibiting the lipopolysaccharide- induced NF-*κ*B/COX-2 signalling pathway and the expression of inflammatory mediators. *Journal of Ethnopharmacology*.

[42] Kumar S., Pandey A. (2013). Chemistry and biological activities of flavonoids: as overview. *The Scientific World Journal*.

[43] Kim H., Cho M., Reddy A. (2005). Down-regulatory effect of quercitrin gallate on nuclear factor-*κ*B-dependent inducible nitric oxide synthase expression in lipopolysaccharide-stimulated macrophages RAW 264.7. *Biochemical Pharmacology*.

[44] Rao-manjeet K., Ghosh B. (1999). Quercetin inhibits LPS-induced nitric oxide and tumor necrosis factor-*α* production in murine macrophages. *International Journal of Immunopharmacology*.

[45] Morikawa K., Nonaka M., Narahara M. (2003). Inhibitory effect of quercetin on carrageenan-induced inflammation in rats. *Life Sciences*.

[46] Souto F., Zarpelon A., Staurengo-Ferrari L. (2011). Quercetin reduces neutrophil recruitment induced by CXCL8, LTB4, and FMLP: inhibition of actin polymerization. *Journal of Natural Products*.

[47] Endale M., Park S., Kim S. (2013). Quercetin disrupts tyrosine-phosphorylated phosphatidylinositol 3-kinase and myeloid differentiation factor-88 association, and inhibits MAPK/AP-1 and IKK/NF-*κ*B-induced inflammatory mediators production in RAW 264.7 cells. *Immunobiology*.

[48] Su H., Kim Y., Park Y., Lee H., Kim K. (2014). Anti-inflammatory effects of chlorogenic acid in lipopolysaccharide-stimulated RAW 264.7 cells. *Inflammation Research*.

[49] Chen W., Wu L. (2014). Chlorogenic acid suppresses interleukin-1*β*-induced inflammatory mediators in human chondrocytes. *International Journal of Clinical and Experimental Pathology*.

[50] Rodriguez J., Theoduloz C., Sánchez M., Razmilic I., Schmeda-Hirschmann G. (2005). Gastroprotective and ulcer-healing effect of new solidagenone derivatives in human cell cultures. *Llife Sciences*.

[51] Wu X., Li X., Xiao F., Zhang Z., Xu Z., Wang H. (2004). Studies on the analgesic and anti-inflammatory effect of bornyl acetate in volatile oil from Amomum villosum. *Journal of Chinese Medicinal Materials*.

